# Operative Procedures for Carcinomatous Tumors of the Breast

**Published:** 1893-12

**Authors:** J. McFadden Gaston

**Affiliations:** Atlanta, Ga.


### *ORIGINAL COMMUNICATIONS.*

### OPERATIVE PROCEDURES FOR CARCINOMATOUS TUMORS OF THE BREAST.*

BY J. McFADDEN GASTON, M. D.,

ATLANTA, GA.

The high estimate placed upon the preservation of the mammary gland is attested by the commendable pride of every fair maiden in the development of this part of her organism and by the practical concern of every mother in the due performance of the function of lactation.

Not only from an esthetic standpoint but from utilitarian considerations, the mammary gland is recognized as entitled to a prominent place in the physical structure of woman, and anything which tends to induce diseases of this organ appeals strongly to the physician and surgeon for relief. It becomes us, therefore, to use all diligence in the prophylactic and curative appliances directed to it.

While the mammary gland is liable to injuries of various kinds and to inflammation in connection with its functions, there is a spe-

*Read before the Southern Surgical and Gynecological Association at New Orleans, November 15, 1893.

cial proclivity to disorders of a graver character, characterized by developments of a malignant kind in its structures.

The mammary gland is peculiarly constituted in the human female and its anatomical relations to the lymphatics render it more liable to complications with the neighboring ganglia, under inflammatory processes, than occurs in any other part of the organism. There are physiological sympathies of this gland with other viscera which indicate a deep seated and intimate correlation between them, but we are interested now especially in the investigation of the link which binds this structure to the general physical system. The involvement of the mammary gland in carcinoma is most frequently associated with marked impairment of the general health, and earlier or later that condition known as cachexia is developed, so as to become characteristic of the progress of a carcinomatous disorder. There is some plausible ground for doubt as to cause and effect in the local and general manifestations of carcinoma, and hence a question in regard to the circumscribed area of the disease at the outset in the tissue of the mammary gland. It is held by most observers that cancer of the breast is primarily a strictly local trouble, and if removed at an early period must be eradicated. But either the tumor has from the outset a proclivity to reproduction, or comparatively few cases are operated on sufficiently early to arrest their progress, as it is well known that a return of the neoplasm is the most frequent result of operations for the removal of carcinomatous tumors of the breast. This holds in regard to the complete as well as the incomplete mode of operating in these cases, and in those patients, without enlargement or induration of the axillary glands, there does not appear any good and sufficient reason for the invasion of this region by the knife. There are no indications of trouble beyond the limits of the breast, and under such circumstances it must prove very difficult, if not quite impracticable, to identify the lymphatic glands and remove them. If it was simply a matter of indifference as to the effect of this complete operation upon the patient, each operator might cut and slash to his heart's content in the axillary and in the clavicular space, but when it adds to the traumatism in a way to damage the prospects of recovery, it certainly should be reserved for the removal of diseased lymphatics. Even in a case of involvement of the ganglia in the clavicular space, it is problematical whether the shock of enucleating them may not overbalance any advantage which can accrue from their removal.

In the legacy left to the profession by Dr. Douglas Morton, in answer to the question, "How should we operate for mammary cancer and when?" it is held that the "completed operation" is not only far more dangerous than the old one, but in an important sense is still incomplete after all. As it is usually done, the axillary glands and some tissues about them are removed. The intervening lymphatic ducts that may be full of cancer cells, especially at their valves, are at best only partially removed; the alveolar buds that shoot out from every gland from the moment it enters the process of becoming fixed are cut or torn through, causing cancer cells to be scattered over the surface of the wound soon to become fixed as new foci. Furthermore the supra and sub-clavicular glands that in many, perhaps most of the cases, become infected sooner or later, are in the "completed operation" as it is ordinarily done, left intact; even Gross, a most earnest advocate of this operation, removes these glands only when they are palpably enlarged. Butlin calls it a "surgical blunder."

In order to examine systematically the conditions that bear upon "clearing out" the axilla, Dr. Morton claims that all mammary cancer subjects may be divided into four classes. (1) Those in which the disease has not only infected the axillary glands, but by metastasis or otherwise has involved the internal tissues and organs; (2) those in which these glands have become infected, but in which the disease has gone no further; (3) those in which metastases have occurred without infecting the glands; (4) those cases in which the disease is as yet limited to the mamma. He goes on to say that the cases of the first class are plainly not operable. In those of the second, if clearing out the axilla and removing every gland in the whole neighborhood of the diseased breast and the tissues around them, as well, were to complete the operation as the term implies, it would certainly be justifiable, provided the mortality after this procedure is not too great.

The statistics of local recurrences given by Gross in 1888 are quoted by Morton as follows:

Of 409 cases, partial or total extirpation of mamma, without glands, was done in 96 cases.

Recurrence in or near the cicatrix, 46 cases, being 47.91 per cent.

Recurrence in cicatrix and glands, 31 cases, being 19.79 per cent.

Recurrence in glands alone, 19 cases, being 32.29 per cent.

Of amputation of breast with removal of glands, 313 cases.

Recurrence in or near cicatrix, 235 cases, being 75.08 per cent.

Recurrence in glands alone, 38 cases, being 12.14 per cent.

Recurrence in both places, 40 cases, being 12.77 per cent.

These figures, Morton believes, show the impossibility of doing any operation that deserves to be called "completed" after the invasion of the axilla has taken place. They show, too, that the number of local foci so far from being diminished, is certainly very much increased. These facts, taken with the other very important fact that the immediate mortality after the "completed operation" is double (according to the statistics of Butlin) that after the incomplete, seems to Morton, to utterly condemn the former as a life-saving measure in this class of cases.

In the above division, the third class of cases, in which metastatic tumors have occurred, without antecedent glandular involvement, are clearly not amenable to operation.

The fourth class embraces those cases in which the cancer is as yet strictly local, and hence eradicable by operation.

Morton thinks that after the axillary glands have once become involved it is highly improbable that the disease can be eradicated by any surgical procedure.

A point of great moment, as to the extent of operative precedure, pertains to the leaving of any portion of the mammary gland, when only partially implicated in the carcinomatous growth. The esthetic element should never enter into the decision of such a vital question as the arrest of carcinoma, and whenever a breast is the seat of a malignant tumor, whether wholly or partially involved, there should be no hesitation about removing the entire glandular structure.

If a part of the mammary gland only seems to be involved and it is evident the knife can be carried outside of the neoplasm into the apparently sound tissues of the breast, there is every reason to believe that if any portion of the gland is left it may become the seat of disease and that recurrence will most likely follow the operation. On the other hand, an entire ablation offers better prospects of success.

The preservation of such part of the skin as may serve to cover up the denuded surface of the thoracic wall, after removal of the breast depends upon the extent to which the cutaneous covering of the tumor is adherent, and consequently involved in the disease. It may be that the direction of the incision will influence materially the capacity for bringing the edges of the skin together, owing to the looseness of the attachment and the elasticity of the tissues in one direction being greater than in another so as to favor traction. In most cases saliency of the tumor admits of making a double elliptical incision, so as to take away the adherent cutaneous covering with the diseased breast, and all the skin which is loosely attached by cellular tissue may be safely preserved to cover the wound without undue traction. Retentive ligatures, at the distance of an inch or more from the margin, may be inserted at the points of greatest tension, and between them broad strips of adhesive plaster may be placed to secure union of the edges. If cancer of the breast returns after removal it is inculcated by most writers to operate again and again, so often as the growth appears, and it is held that thus the life of the patient is prolonged. At the present day, when a patient is free from suffering by an operation, under the influence of anesthesia, there can be no objection to the repetition of cutting while the patient is disposed to submit to the knife. But a patient whose breast was removed in a carcinomatous state some three and a half years ago, had a return of the disease within two years, and took matters into her own hands by employing a quack to treat it with escharotics. I had occasion to examine this lady since, and if she was correct in her conclusion that there was a redevelopment of the cancer, it must be allowed that benefit has resulted from the escharotic treatment. The healing process has proceeded so that all the wound is completely cicatrized.

The object lesson afforded by the good result in the case has served to gain another convert to escharotic treatment, in the person of another patient, upon whose breast I had performed a primary operation and a colleague had done a secondary operation for a recurred tumor.

Being impressed with the failure of the knife to avert the carcinomatous growth, the husband of the lady acquiesced in her desire to submit to the escharotic treatment on the occasion of the reproduction of the tumor a second time. In my operation the mammary gland was removed, but finding no involvement of the axillary glands, this region was left untouched. When the second operation was determined upon, in my absence from the city, the colleague upon whom it devolved found an enlarged lymphatic gland near the anterior axillary fold, which was removed by the extension of his incision from the site of the tumor, so as to include this gland.

This complete operation did not secure any better result than my incomplete operation had done, and within a very few months I was called again to verify, not alone the return of the tumor, but also the development of the gland. My opinion was requested as to these growths, which were pronounced to be indications of an unmistakable kind, that the carcinomatous disease had not been eradicated. It was also stated that, in view of the past failures with the knife, little was to be expected from a third cutting operation for the extirpation of the reproduced tumor and gland, and the husband was left to his own choice in selecting the treatment of escharotics on this occasion.

The great suffering inflicted upon the patient by the employment of escharotics in the treatment of malignant growths is an objection of great moment, and yet with the use of morphine the pain is so far mitigated as to become bearable. In a recent treatment of epithelioma of the nose, advanced to the ulcerative stage, involving the ala and septum, the pain from destructive action of the Vienna paste at the outset and the arsenical paste subsequently, required the free use of hypodermics of morphine and atropia to enable the patient to submit to their applications. He bore the pain in the spirit of martyrdom, as I had advised removal with the knife and then to perform a plastic operation immediately to rebuild the nose from the tissues of the cheeks. Upon his urgent request to have the escharotic used, I resorted to this mode of treatment under protest, telling him that it would leave him greatly disfigured to effect a thorough eradication of the cancer. He seems to be now free from disease after the destruction of almost the entire nose.

The relative advantages of the knife and cauteries in the management of carcinoma depend very much upon the progress of the disease. In the incipiency of the local trouble there can be no doubt in regard to the excision being preferable to cauterization, but after full development of a tumor with a tendency to degeneration and breaking down of its structure the resort to escharotics has its advantages in extending to the remote ramifications of the disease. It is a prevalent impression that certain caustic applications attack the diseased structure without affecting the sound tissues and that the so-called roots of a cancer are thus destroyed. There seems to be some just foundation for this belief in regard to application of arsenic, but the destructive effect of caustic potash in the form of Vienna paste extends to every vital structure with which it comes in contact, and the same holds in reference to the plaster of sulphuric acid and charcoal as an escharotic.

The chloride of zinc application is not accompanied with so much pain as the previous named escharotics, and yet does not prove so effective, owing to its less destructive agency.

Quite a number of vegetable products have been likewise employed for the treatment of cancerous affections, but are not so prompt and radical in their effects as the chemical escharotics, and, as a consequence, require a longer time to bring about the disintegration of the morbid growth.

One of the most serious obstacles to the progressive improvement of caustic means applied by so-called doctors is the infatuation that their interests are best served by secrecy as to the remedies used by them. The people who seek their advertised treatment are imposed upon by the air of mystery thrown around their procedures, and if the facts were known, it would appear clearly that there was no virtue in their drugs.

The treatment of carcinomatous tumors of the breast with caustics has been tested fully by Bougard of Belgium. His paste contains chloride of zinc, arsenic, cinnabar and corrosive sublimate. Of one hundred and sixty cases, sixty-two, or nearly forty per cent., were free from recurrences three years after treatment.

Bougard's experience leads to the inference that there is something connected with the escharotic application in cases of carcinoma, which is more pervading and far-reaching than simple excision with the knife. As this cauterization does not look to the dissection of the glands from the axilla or clavicular region, it seems that this is not requisite to secure the most favorable results.

Having received a circular letter some two months ago, embodying interrogations upon various points, connected with cancer of the breast, it may not be out of place to incorporate them with my replies.

1. Have you ever seen a breast cancer heal spontaneously? Answer.—No.

2. Have you ever lost a case as the direct result of operation, since the advent of antiseptic methods? Answer.—Yes.

A single case of encephaloid cancer of the breast of large proportions and necessitating extensive exposure of the thoracic wall, died from shock six hours after complete ablation of the mammary gland.

3. About what is the expectation of life after operation? Answer.—This depends to a large extent upon the period at which an operation is undertaken; those done early, recover, and those delayed rarely escape a fatal termination.

4. Have you any case of survival of operation without recurrence longer than three years? Answer.—One case was operated upon nearly five years ago, who has had no return of disease; and is in good health at present. The entire breast was removed without entering the axilla.

5. Have you any cases of survival of operation for three years, and later development of cancer? Answer.—No.

6. About how many cases have you operated upon? Answer.—Ten cases, within the past ten years.

7. Has it been your invariable practice to remove the axillary glands, at the time of operation, or merely in cases when there was evidence of involvement? Answer.—The axilla has not been opened except when there was evident enlargement and induration of the lymphatic glands; and my observation of a departure from this course by others has not proved satisfactory. There is ground for doubt as to the advantage of removing the lymphatic glands even when involved in the disease.

